# A mixed-method feasibility study of the use of the Complete Vocal Technique (CVT), a pedagogic method to improve the voice and vocal function in singers and actors, in the treatment of patients with muscle tension dysphonia: a study protocol

**DOI:** 10.1186/s40814-023-01317-y

**Published:** 2023-05-24

**Authors:** Julian McGlashan, Mathias Aaen, Anna White, Cathrine Sadolin

**Affiliations:** 1 Ear, Nose and Throat Department, Queen’s Medical Centre Campus, Nottingham University Hospitals, Nottingham, NG7 2UH UK; 2 Complete Vocal Institute, Kompagnistraede 32A, 1208 Copenhagen K, Denmark; 3 Honorary Researcher, Ear, Nose and Throat Department, Queen’s Medical Centre Campus, Nottingham University Hospitals, Nottingham, NG7 2UH UK

**Keywords:** Muscle tension dysphonia, Voice therapy, Feasibility study, Telehealth

## Abstract

**Background:**

Muscle tension dysphonia (MTD) results from inefficient or ineffective voice production and is the cause of voice and throat complaints in up to 40% of patients presenting with hoarseness. Standard treatment is voice therapy (SLT-VT) delivered by specialist speech therapists in voice disorders (SLT-V). The Complete Vocal Technique (CVT) is a structured, pedagogic method which helps healthy singers and other performers optimise their vocal function enabling them to produce any sound required. The aim of this feasibility study is to investigate whether CVT administered by a trained, non-clinical CVT practitioner (CVT-P) can be applied to patients with MTD before progressing to a pilot randomised control study of CVT voice therapy (CVT-VT) versus SLT-VT.

**Methods/design:**

In this feasibility study, we use a mixed-method, single-arm, prospective cohort design. The primary aim is to demonstrate whether CVT-VT can improve the voice and vocal function in patients with MTD in a pilot study using multidimensional assessment methods. Secondary aims are to assess whether (1) a CVT-VT study is feasible to perform; (2) is acceptable to patients, the CVT-P and SLT-VTs; and (3) whether CVT-VT differs from existing SLT-VT techniques. A minimum of 10 consecutive patients with a clinical diagnosis of primary MTD (types I–III) will be recruited over a 6-month period. Up to 6 video sessions of CVT-VT will be delivered by a CVT-P using a video link. The primary outcome will be a change in pre-/post-therapy scores of a self-reported patient questionnaire (Voice Handicap Index (VHI)). Secondary outcomes include changes in throat symptoms (Vocal Tract Discomfort Scale), acoustic/electroglottographic and auditory-perceptual measures of voice. Acceptability of the CVT-VT will be assessed prospectively, concurrently and retrospectively both quantitatively and qualitatively. Differences from SLT-VT will be assessed by performing a deductive thematic analysis of CVT-P transcripts of therapy sessions.

**Conclusion:**

This feasibility study will provide important data to support whether to proceed with a randomised controlled pilot study focusing on the effectiveness of the intervention compared to standard SLT-VT. Progression criteria will be based on demonstrating a positive outcome in treatment, successful delivery of the pilot study protocol, acceptability to all stakeholders and satisfactory recruitment rates.

**Trial registration:**

ClinicalTrials.gov website (NCT05365126 Unique Protocol ID: 19ET004). Registered on 06 May 2022.

**Supplementary Information:**

The online version contains supplementary material available at 10.1186/s40814-023-01317-y.

## Background

Hoarseness and associated vocal problems (dysphonia) cause both a major impact on quality of life and livelihood for affected patients as well as posing a substantial healthcare burden [1]. Approximately 40% of patients referred for assessment of hoarseness will be diagnosed with primary muscle tension dysphonia (MTD), a condition causing voice impairment and/or reduced vocal capacity in the absence of known structural, neurological or inflammatory laryngeal pathology [2, 3]. Other symptoms of MTD may include vocal fatigue, vocal strain, aberrant pitch and pitch range, variability in the quality or control of voice, difficulties in voice projection, reduced vocal stamina and vocal tract discomfort [4].

**Fig. 1:**
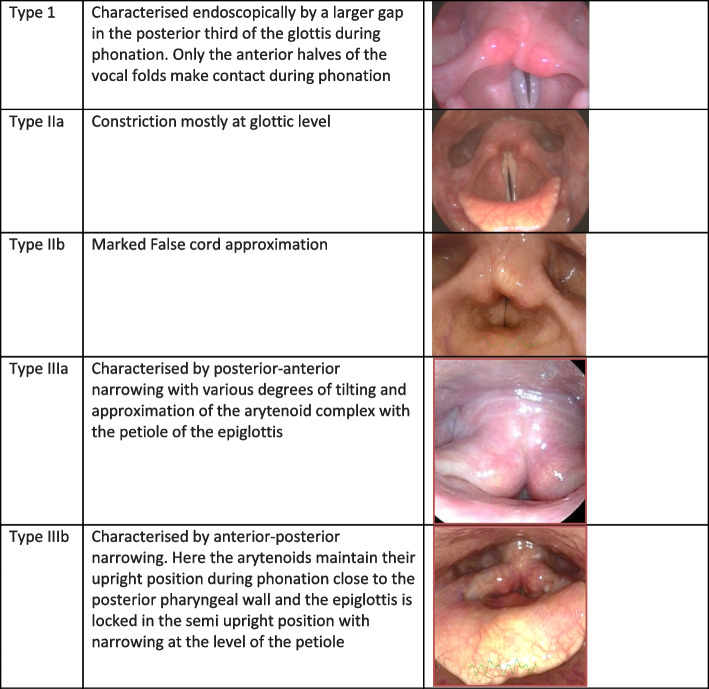
Endoscopic appearance of the larynx demonstrating MTP patterns types I–III

Primary MTD is thought to arise from dysregulated or imbalanced laryngeal and para-laryngeal muscle activity due to dysfunctional prefrontal cortical regulation that may interfere with laryngeal motor preparation, initiation and execution combined with heightened input from limbic regions [2, 5, 6]. This results in excessive contraction of extrinsic laryngeal, suprahyoid and/or strap muscles which tend to elevate the suspended hyoid-larynx complex and in turn sympathetically induces intrinsic laryngeal hyperfunction [7]. This laryngeal hyperfunction is often associated with dysfunctional breathing patterns and use of resonance [8]. Secondary MTD is compensatory behaviour to overcome organic pathology [9]. There is no objective diagnostic test for MTD. A diagnosis of primary MTD is usually made on (1) clinical history, presenting symptoms and voice quality; (2) absence of organic pathology and (3) the appearance of the larynx on laryngostroboscopy [6, 10]. Although numerous classifications of MTD have been described in the past, six recognisable patterns of primary MTD are often described [6, 11, 12]. Three types (MTD patterns I–III) are more related to ineffective voice use, also known as ‘voice abuse’ or ‘voice misuse’, which will be the focus of this study (Fig. 1). The other three types (MTD patterns IV–VI) have a predominantly psychological basis [13–18] although personality and/or psychological issues may also contribute to perpetuation of abnormal MTD patterns in types I–III in some cases [19].

### Standard treatment of MTD

Traditional treatment of MTD consists of indirect voice therapy and direct voice therapy [20] given by a speech and language therapist specialised in voice disorders (SLT-V) [21, 22]. Indirect voice therapy consists of education, information, vocal hygiene and psychological support to encourage behavioural change [23] whereas direct voice therapy consists of establishing healthy voice production [24, 25] by rebalancing the three subsystems of voice production namely breathing (respiration), voice production (phonation) and more efficient use of resonance [8, 26, 27]. Direct voice therapy however is not just one treatment method, and many SLT-Vs use a hierarchical and experiential approach drawing on multiple techniques but often with limited evidence to support their adoption [28]. Problematically, voice therapy has been portrayed as a ‘black box; and many recognised treatment regimens overlap in their aims and therapeutic goals with often limited descriptions of ‘active ingredients’ [20, 28, 29]. In addition, outcome measures are used inconsistently and there are limited reports on treatment fidelity [28]. This makes it difficult to determine why patients improve, which therapy tasks are most beneficial and for how long they should be given [20, 28, 30–33]. There are also few studies looking at the long-term benefits of all voice therapy modalities [7].

### Use of the complete vocal technique in vocal performers

The complete vocal technique (CVT) is pedagogic technique primarily used by singing teachers and vocal coaches to aid singers and actors produce the vocal sound and vocal function that the performer requires [34] and has been used for over 35 years, particularly in Europe. CVT practitioners (CVT-P) undergo an accredited 3-year training programme to achieve competency. CVT uses a systematic approach with terminology that is clearly defined and supported with scientific characterisation [35–38]. Specific elements of CVT have been packaged together in what is termed CVT voice therapy (CVT-VT) which is used for performers presenting with acute vocal problems. Although CVT-VT has been used primarily to improve or rehabilitate the singing voice it has also been used to help the performing speaking voice but not in a patient population. In addition, there have been no studies on CVT-VT and no comparison has been made between standard SLT-VT and CVT-VT techniques. It is unclear what, if any, differences there are in the ingredients and targets [39].

### Use of a video link for delivery of therapy and instruction

Traditionally, voice therapy for patients, and CVT training for performers, has been given face-to-face. However, with the onset of the COVID-19 pandemic, SLT-VT and CVT training have almost entirely been given using telehealth via a video link [40]. There are few randomised controlled studies comparing face-to-face versus telehealth for voice disorders, and only one study in MTD patients, which showed no significant difference in outcome between the two methods of delivery [41]. To our knowledge, there has been no evaluation of singing instruction, including CVT, given by a video link to performers delivered by vocal coaches.

### Potential benefits and harm of using CVT-VT in MTD patients

Vocal performers need rapid return to professional vocal function to avoid loss of employment, income and reputation. CVT-VT is used by CVT-P’s for this purpose but there is only anecdotal evidence of its effectiveness in the acute situation. CVT-VT has the potential benefit of providing rapid improvement in MTD patients as it is based on a well-defined, systematic assessment and evaluation protocol [34]. There is no evidence that patients come to serious harm from SLT-VT interventions for MTD or singers from CVT-VT, but to our knowledge, there are no studies addressing this specific issue. A small longitudinal study of the vocal health of twenty singers using CVT over 14 years however did not show any detrimental effects [42]. Adverse outcomes with SLT-VT are mostly reported as ‘no improvement’, ‘non-compliance’ or patient ‘drop-out’ [7], and it is possible the same may apply to patients treated with CVT-VT.

### Rationale and aims for the study

Patients with MTD and vocal performers both present with voice impairment and/or reduced vocal capacity due to dysregulated or imbalanced laryngeal and para-laryngeal muscle activity associated with dysfunctional breathing patterns and inefficient use of resonance during phonation. CVT-VT administered by appropriately trained practitioners provides a well-described tool that could potentially be used to restore vocal function in MTD patients. Although CVT-P’s are highly trained, their clientele are largely healthy vocal performers and traditionally they refer clients with unhealthy voices for clinical assessment and treatment. The aim of this feasibility study is to address the following uncertainties before proceeding with a pilot randomised clinical trial of CVT-VT versus SLT-VT: (1) Can CVT-VT applied by a CVT-P improve the voice and vocal function in patients with MTD? (2) Is it feasible to perform a pilot study using CVT-VT administered by a CVT-P using telehealth? (3) Is CVT-VT acceptable to patients, CVT-Ps and SLT-VTs? (4) Does CVT-VT offer a new approach to improving the voice and vocal function compared to traditional SLT-VT methods? Progression criteria will use the traffic light system [43, 44] and be based on demonstrating a positive outcome in treatment, acceptability to all stakeholders and successful delivery of the pilot study protocol.

## Methods

### Study design

This prospective feasibility study [45] of CVT-VT for MTD has a mixed-method, non-randomised, single-arm design. It will be conducted in a tertiary voice centre in Nottingham, UK. The study protocol has been approved by the NHS Health Research Authority (HRA) and Health and Care Research Wales (HCRW) (14 April 2022) following favourable Ethical opinion for conduct by the East of England—Cambridge South Research Ethics Committee (Reference no. 22/EE/0047). The study will be reported in accordance with the CONSORT extension to pilot studies [46] and SPIRIT guidelines [47] as outlined in Thabane et al. [48].

### Participants and setting of the study

**Fig. 2:**
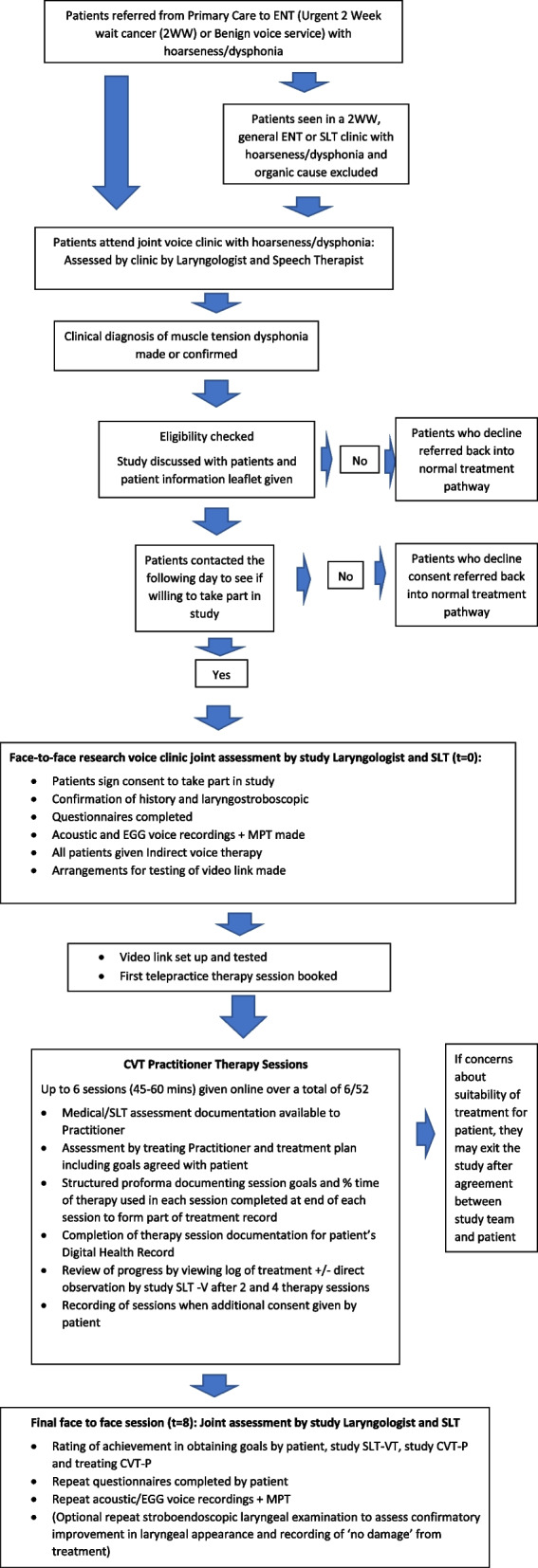
Flow chart (V2.0 28 Feb 2023)

Patients referred by Primary Care Physicians to the Ear, Nose and Throat (ENT) Department, Queen’s Medical Centre Campus (QMC), Nottingham University Hospitals, with hoarseness will be screened and those with a clinical diagnosis of MTD will be reviewed in a joint voice clinic in the ENT Department, for more specialist assessment. A diagnosis of primary MTD will be made by consensus between an experienced laryngologist and SLT-VT following detailed medical evaluation including laryngostroboscopic examination. Consecutive patients with a confirmed diagnosis of MTD due to 'voice abuse/misuse' (Table 1) who meet the inclusion (Table 2) and exclusion criteria will be invited to take part in the study and at least 10 will be recruited (Fig. 2).

**Table 1:** Symptoms and clinical findings compatible with a clinical diagnosis of MTD

•The history of the presentation of the condition and its compatibility with a Primary MTD diagnosis
•Presenting vocal symptoms such as hoarseness, change in voice quality, limitations in pitch, loudness, flexibility, and/or stamina of the voice
•Absence of organic pathology such as structural abnormalities, neurological and inflammatory conditions on endoscopy
•Auditory-perceptual voice change which includes one or more of the following characteristics: having a variable or abnormal in quality (overall severity of hoarseness, roughness, breathiness, strain), having an abnormal habitual pitch with or without a restricted fundamental speaking frequency range or having abnormal loudness and loudness variability during speech
•The presence of muscle soreness, tenderness or other evidence of hyperfunction in the thyrohyoid or cricothyroid space and/or suprahyoid muscles on physical examination
•Laryngoscopic findings of laryngeal hyperfunction pattern (MTP types 1–III)

**Table 2:** Inclusion and exclusion criteria

**Inclusion criteria**
•Males and females
•18 or above
•Clinical diagnosis of primary MTD based on history and laryngoscopic assessment (type I–III MTD patterns) through joint assessment by a SLT-V and laryngologist
•Current voice problems, persistent for greater than 2 months
•Severity of disorder (a) VHI ≥ 30 and (b) patient wants therapy
•Patient willingness to undergo treatment
•Agree to undertake the study protocol
**Exclusion criteria**
•Organic vocal pathology (1) structural/neoplastic disorders (e.g. carcinoma, cyst, polyp, papilloma, Reinke’s oedema), (2) neurological disorders (e.g. vocal cord palsy, paresis, spasmodic dysphonia) and (3) inflammation (e.g. infection, reflux (RFS > 7) or significant relevant systemic disease (e.g. severe COPD) or need for surgery
•Significant psychological issues identified during initial assessment (with option to withdraw if discovered during the treatment periods and agreed by both patient and therapist)
•MTD pattern (IV–VI) compatible with significant primary psychological aetiology on laryngoscopy
•Transgender voice issues
•Previously incompletely treated dysphonia, neurological disease or upper aerodigestive tract malignancy
•Had previous VT or CVT training or pharmacological treatment for their voice problem (other than proton pump inhibitors or an alginate recommended for disorders of laryngopharyngeal reflux–related symptoms)
•A hearing impairment that would prohibit or impact on telepractice treatment
•Significant concomitant health problems affecting voice
•Not have or be able to use a computer with video link at home or in hospital even with support
•Not able to commit to the study protocol

### Recruitment and consent

#### Recruitment voice clinic

All patients attending the voice clinic in the ENT department at NUH will be assessed by a laryngologist and SLT-VT and have a nasoendoscopic laryngostroboscopic examination prior to recruitment to rule out organic pathology and confirm the pattern of muscle tension dysphonia. Patients identified as having primary MTD seen will have their eligibility checked and given a patient information sheet outlining the study. They will be contacted by phone after their clinic appointment to see if they are willing to consent to taking part. Those interested will be invited to attend a research clinic run by the study laryngologist and SLT-VT. Those who decline will be referred on the standard SLT voice therapy pathway.

#### Research clinic and consent

Patient eligibility will be checked, and written consent obtained. Study questionnaires including a goal-setting checklist will be completed. Acoustic and EGG recordings will be made including MPT and the case-report form (CRF) completed. Prior to commencing therapy, all participants will be given indirect voice therapy in the form of a vocal hygiene advice leaflet (see Additional file 1) when they attend the Research clinic. A post-visit check of the participant’s video link will be made prior to the start of the therapy sessions. Following completion of the CVT-VT, participants will be asked to attend a further Research Clinic appointment for post-therapy assessment (*t* = 8), feedback on their experience, and whether goals for treatment were met. Should further therapy be required, this will be arranged in the form of standard SLT-VT.

### Interventions

#### CVT-VT sessions

The therapy provided will be reported in accordance with the TIDieR guidelines [49]. Patient contact details will be passed on to the CVT-P who will make contact with the patient by email or mobile to arrange video therapy sessions. These will be conducted using the NUH Trust-approved platform DrDoctor. Prior to the first therapy, the CVT-P will review all the patient information (summary case history and clinical findings, voice and EGG recordings, and laryngostroboscopic video recordings). Up to 6 forty-five-minute therapy sessions will be arranged between the CVT-P and patient within an 8-week period. An additional 15 min/session will be allowed for resolving connection, technical, patient and therapist attendance issues.

#### CVT-VT healthcare intervention

The therapy will consist of exercises to learn sufficient control over the support system, exercises for obtaining prototypical phonation types based on the volume and quality requirements from the patient, along with additional exercises for dynamic control over loudness, colouring of voice and for expressivity. The therapy uses a systematic building of skills starting with voiceless support exercises, progressing to the ability to connect support to sustained vowels, towards speech and voicing tasks including pitch change and consonants, building into phrases and sentences. Dependent on the patient’s symptoms, goals and desired sound character(s), specific exercises for appropriate and healthy use of the CVT specific vocal modes that correspond to the desired loudness and quality will be chosen. For low volume or breathy voice symptoms, a more twanged and more intentionally narrowed epilaryngeal space is sought. For hyperfunction symptoms, adjustments in either breath control or releasing of unintentional constriction is sought to counter the hyperfunction and related pressed voice. Moreover, stamina and longevity are addressed by exercises focusing on sustained healthy voice use. CVT techniques and tools addressing the specific needs and impairments of each individual patient will be documented in qualitative notes, allowing for subsequent analysis of the employed intervention strategies. Moreover, patients will be asked to maintain the exercises between therapy sessions, with the CVT-P inquiring about progress between sessions at initiation of every therapy session. The outline of the therapy sessions is listed in Additional file 2.

#### Objectives and outcomes

The primary aim of this study is to demonstrate that CVT-VT is beneficial to patients with primary MTD. Secondary aims include (1) whether it is feasible to perform a pilot study using CVT-VT administered by a CVT-P using telehealth; (2) whether CVT-VT is acceptable to patients, the CVT-Ps and SLT-VTs; and (3) whether CVT-VT differs from SLT-VT. The outcomes will provide important evidence for the feasibility of planning a larger pilot randomised controlled trial focusing on the effectiveness of the intervention compared to standard SLT-VT.

#### Primary aim: does CVT-VT delivered by a CVT-P improve the voice and vocal function in patients with primary MTD?

This question will be addressed using a prospective, non-randomised, single-arm pilot study using a range of multidimensional measures. The study will also test the utility of some of these measures in this patient population by measuring changes in pre- and post-treatment values and provide initial data for the primary outcome measure, to help determine a sample size calculation for a larger trial [50].

Treatment improvement will form one aspect of the evaluation of feasibility to use CVT-VT delivered by a CVT-P to treat patients with muscle tension dysphonia. The objectives are to determine whether CVT-VT is beneficial in the treatment of patients with primary MTD using both patient-rated and physician-measured outcome measures to give a multidimensional assessment of voice and vocal function outcome pre- and post-treatment. Although there is general agreement on some outcome measures such as patient self-evaluation questionnaires, there is less consensus on the auditory-perceptual and objective measures of voice such as acoustic, electroglottographic (EGG) and aerodynamic measures.

The patient measures will include two validated disease-specific patient self-evaluation questionnaires the Voice Handicap Index [39, 51] and the Vocal Tract Discomfort Scale [42]. These measures assess a range of psychosocial, physical voice impairment symptoms, the ability to be heard and throat symptoms [52]. As this study is designed to determine feasibility rather than effectiveness, an improvement in the median score of the VHI of > 20% for the group will be taken as a positive outcome. The physician measures will include a range of acoustic, electroglottographic (EGG) and aerodynamic measurements as well as an auditory-perceptual evaluation of the voice using the CAPE-V rating scale [53].

#### Primary outcome measure: Voice Handicap Index (VHI)

Patient-reported outcomes are generally accepted as the most relevant tool for evaluating treatment effectiveness of voice disorders as they may provide a more a more meaningful impact overall of a voice disorder [52, 54]. Of these, the validated VHI [51] has been shown to have one of the best psychometric properties among voice-specific quality of life instruments [55, 56] and will be the primary outcome measure for this study. The VHI has been validated with strong internal consistency and test–retest reliability and has been used as a functional outcome measure from behavioural voice treatment in clinical practice and in clinical research which allows cross-study comparisons of treatment response [51, 57–60]. VHI scores can range from 0 (no ‘handicap’) to 120 (maximal ‘handicap’), with scores below 30 generally associated with minimal ‘handicap’. Thirty was one of the eligibility criteria used for the study. Changes in VHI scores of 18 points or greater from pre-treatment to post-treatment have been reported to indicate a meaningful clinical effect [51].

#### Vocal tract discomfort scale (VTDS)

Although voice problems are the most common presenting complaint in patients with MTD, many have associated symptoms of vocal tract discomfort [53]. It is thought these relate to increased vocal effort and vocal fatigue [53, 61, 62]. The VTDS is a validated self-administrated questionnaire designed to measure the subjective perception of sensory discomfort in the throat (vocal tract) [53]. Patients are asked to rate the frequency of occurrence and severity manifestation of eight subjectively different sensations: burning, tightness, dryness, aching, tickling, soreness, irritability and lump in the throat. The frequency and severity are rated separately on a seven-point Likert scale ranging from 0 to 6 for frequency (0 = never, 2 = sometimes, 4 = often, 6 = always) and for severity (0 = none, 2 = mild, 4 = moderate, 6 = extreme) [63]. VTDS scores have been shown to correlate with the total and physical domain score of the VHI and decrease after voice training and vocal hygiene education in teachers [64]. A change in the Persian version of the VTDS of 6.0 points for each subscale following a therapeutic intervention has been interpreted as a real change with a 95% confidence level [65]. Again an improvement in the median score for the group will be taken as a positive outcome.

#### Acoustic and EGG measures

Pre- and post-treatment acoustic and EGG measurements during phonation provide an objective assessment of the different aspects of the voice and vocal function at specific points in time. There are no universally agreed parameters to measure and so it is common for a range of individual and combined multidimensional parameters to be extracted from both sustained vowels and read text. The quality and standardisation of the recordings is important to avoid measurement error. To that end, the acoustic and EGG measures will be recorded in a treatment room, which exhibits nominal ambient room noise. The recordings will be made at the time of the research clinic appointments (*t* = 0, *t* = 8) using a head-mounted omnidirectional microphone placed approximately 3 cm from the left side of the corner of the mouth. The synchronous EGG signal will be recorded from two electrodes placed on either side of the larynx of the subject and inputted into the laryngograph microprocessor. The signals will be processed using Speech Studio software program (vers. 5.21. Laryngograph® Ltd.) and saved as .wav files. The recordings will then be analysed using the in-built statistical programs and into the computer program Praat [66] (Paul Boersma & David Weenink, Institute of Phonetic Sciences, University of Amsterdam, the Netherlands; http://www.praat.org/).

The following voice parameters based on EGG and acoustic measures reflecting different aspects of vocal function will be assessed:

1. Sustained vowels

Acoustic and EGG signals recordings will be made of the four English corner vowels as in the words: /i:/ as in feet, /u:/ as in food, /æ/ as in fad, /α:/ as in farm. They will be analysed using the multidimensional voice profile (MDVP) analysis programme in Speech Studio (vers. 5.21. Laryngograph® Ltd.) [67]. The multidimensional measures will include the average fundamental frequency (Fx), average vocal fold contact quotient (Qx) and mean sound pressure level (mean SPL); a range of perturbation measures (standard deviation of the fundamental frequency (SD of Fx), standard deviation of the contact quotient (SD of Qx), jitter factor, shimmer dB, cepstral peak prominence (CPP), relative amplitude perturbation (RAP)); and measurements of spectral noise energy versus harmonic energy (normalised noise energy (NNE), harmonics to noise ratio (HNR)) [67]. Spectral energy measurements have been reported to be the most correlated acoustic measure with perceptual judgments of roughness, breathiness and hoarseness. It is hypothesised that perturbation and spectral noise energy versus harmonic energy measures will improve following therapy.

The Praat computer program will be used to calculate CPPS, the AVQI and H1-H2 ratio from the recordings [66]. Unidimensional cepstral acoustic measures such as cepstral peak prominence (CPP) [68] and smoothed CPP (CPPS) [69] have been used as reliable predictors for dysphonia with values reducing as dysphonic severity increases [70]. It has been found to have better sensitivity, specificity, and positive and negative predictive values than jitter, shimmer and NHR. CPP is the prominent peak with the highest amplitude representing the fundamental frequency. CPPS has also been shown to be a good measure of vocal fatigue in patients with hyperfunctional voice disorders [71]. Acoustic measurement of smoothed cepstral peak prominence (CPPS will be made on the stabilised 1 s mid-portion of sustained /a/ vowel (CPPs-/a/) and the CAPE-V voice phrase ‘We were away a year ago’ phrase (CPPS-s).

In addition, the mean level difference in decibels (dB) between the first and second harmonics (H1-H2) of all voiced segments will be measured for the vocal tasks. H1-H2 is a low-bandwidth measure of spectral tilt that provides an estimate of vocal fold closure during phonation [72]. Less abrupt/reduced vocal fold closure associated with a breathier voice quality is reflected in larger differences between the two harmonics (higher H1–H2); conversely more abrupt or increased vocal fold closure is associated with a more strained voice quality and smaller differences between the two harmonics (lower H1–H2) [73–75]. H1-H2 differences could therefore provide an additional measure of change in glottal contact in response to therapy [76].

2) The sentence “We were away a year ago” (from CAPE-V)

Other comparative pre- and post-treatment spectral analyses will also be performed using long-term average spectra (LTAS). LTAS is a fast Fourier transform-generated power spectrum of the frequencies comprising a speech sample. Thus, the LTAS is a composite signal representing the spectrum of the glottal source as well as the spectrum or resonant characteristics of the vocal tract. LTAS holds promise as an acoustic index of voice quality [77]. For example, relatively weak harmonic energy in the higher frequencies of the speech spectrum and a corresponding increase in spectral tilt are characteristic of breathy or hypo-functional signals [68, 78]. In contrast, excessive vocal fold impact and turbulent noise, both of which have been noted in functional dysphonia, are associated with relatively greater energy in the higher frequencies of the speech spectrum [79]. The Sentence “We were away a year ago” features all voiced phonemes and provides a context to judge possible voiced stoppages/spasms and one’s ability to maintain voicing from one word to another [64].

3) A passage of read text ‘Arthur the Rat’

It has been argued that connected speech is a better reflection of vocal function compared to sustained vowels [67]. A range of statistical measures based on EGG and acoustic measures of connected speech in Speech Studio™ describe different aspects of vocal fold vibration and function [67, 80]. These include the following:

- Mode speaking fundamental frequency (Fx) (Hz) + coherence %
- Mode Loudness level (Ax) (dB) + 80% dB range + coherence %
- Mode contact quotient (Qx) % + 80% contact % range + coherence %
- Irregularity scores Fx, Ax, Qx (%)
- Speech range profile (80%)
  - ◦ 80% minimum and maximum intensity (dB)
  - ◦ 80% maximum—minimum frequency range converted to Semitones (Semitone range)

4) Sustained vowels and vowels from speech samples: acoustic formant frequency measures of vocal tract length and formant space

There is radiographic evidence that individuals with vocal hyperfunction exhibit a significantly higher laryngeal position and reduced hyolaryngeal space with consequent shorter vocal tract lengths (VTL) than individuals with healthy voices [81–83]. Raising of the larynx is a consequence of increased extrinsic laryngeal muscle activation, specifically, activation of the thyrohyoid, digastric, stylohyoid, mylohyoid, geniohyoid, hyoglossus and/or genioglossus muscles [84]. Changes in VTL cause a change to all formant frequencies, with a shorter VTL corresponding to increased formant frequencies [82, 85, 86]. A simple relationship between VTL and formant frequency can be derived by modelling the vocal tract as a uniform tube that is closed at one end and open at the other, which exhibits odd quarter-wave harmonic resonances [86, 87]. More reliable estimates can be made using higher formant frequencies (F3 and F4) as they are more stable [88, 89]. A high larynx can lead to restricted tongue movements and vocal tract shaping which can impact on clarity of vowel formation (balance between F1 and F2 formants). It has been shown that formant frequencies for corner vowels are dependent on multiple subject and phonetic context factors [84] but within subject changes secondary to therapy for example could be potentially detectable if all other factors are kept constant. Changes in the formant frequencies from the sustained vowels and from the extracted corner vowels from stable parts of the read passage (‘Arthur the rat’) will be compared pre- and post-therapy.

5) Happy birthday to you

This song has been chosen as it is one of the most widely recognised songs in the English language. However, it is technically quite difficult for non-trained singers as it has a high note, an octave higher, (or seven note jump in the musical scale) than the starting note and small intervals that are near each other. Although reaching the top note does depend on the starting note, it is a reasonably good measure of the flexibility of the voice. The proposal is to perform acoustic and EGG recordings whilst singing the four lines. Each line will be analysed for changes in the LTAS spectral slopes pre- and post-treatment.

#### Auditory-perceptual evaluation

Auditory-perceptual evaluation of voice by expert trained listeners is a subjective judgment on the type and severity of the dysphonic quality present [90]. No auditory-perceptual rating is perfect [91, 92], but CAPE-V is widely used in both the clinical and research settings as it provides a finer judgement of voice quality than the four-point ordinal scale used in GRBAS [93]. CVT have developed their own speech assessment rating using CVT-specific terminology and will also be used.

1. CAPE-V

The Consensus Perceptual Auditory Evaluation of Voice (CAPE-V) is a standardized clinician-made auditory-perceptual measurement of voice that provides an overall rating of severity as well as discreet ratings of specific vocal parameters including overall severity, roughness, breathiness, strain, pitch and loudness [94]. In this study, pitch and loudness will be omitted to reduce rater fatigue and as it can be more reliably assessed using EGG/Acoustic measures. The voice samples to be assessed will consist of the first paragraph of ‘Arthur the rat’ and sustained vowels. Training and external anchors will be provided to overcome the reduced intra-rater and inter-rater agreement associated with the increased freedom of judgement [92].

All voice samples will be rated by four experienced SLT-Vs who will undergo a brief refresher training programme in the use of CAPE-V to improve inter-rater reliability [95, 96]. The raters will be blinded to the whether the recording is pre- or post-therapy. Twenty percent of samples will be re-rated. Samples will be randomly ordered and coded. The severity of each judgment will be quantified by an ‘X’ mark through a 100-mm horizontal line, where the far left end of the line represented normal (and thus assigned a rating of 0) and the far right end of the line represented most abnormal (and thus assigned a rating of 100) [94, 97, 98]. Listeners will rate the perceptual dimensions of (a) overall severity, (b) roughness, (c) breathiness and (d) strain in a similar manner as that of Kapsner-Smith et al. [99]. The mean rating of the four judges for each recording will be used as the data point for individual patients.

2) CVT speech therapy assessment rating (CVT-STAR)

The CVT speech assessment rates three overall parameters: (1) descriptive technical parameters, (2) additional speech parameters and (3) parameters for detecting issues (see Additional file 3). All parameters are rated on a 3-point scoring. The scale ratings are 0 = not at all, 1 = mild, 2 = moderate and 3 = a lot/much. Any speech assessment involves at least (1) the technical parameters and (3) the detection of issues. The additional speech parameters (2) are assessed if deemed necessary by the CVT-P. The three parameters are and include the following:

- **Descriptive technical parameters **include descriptions of mode of vibration (mainly), the amount of metallic character in the voice, the degree of density in the voice and the chosen vocal mode and vocal mode variation, whether the speaker is within the centre of the chosen vocal mode, to what degree there are vocal effects present, to what degree there are rough vocal effects present and degrees of voice instabilities and degree of strain.
- **Additional speech parameters** include rating of sound colour, amount of twang, the speed of speech, the pitch, pitch variation, accentuation/stressing of words, volume and size of the larynx.
- **Parameters for detecting issues** include rating the degree of support energy/effort, the degree of economising breath, assessment of inhalation, the opening of the mouth and a final conclusion describing the assessed main issue to be addressed.

All voice samples will be rated by four experienced CVT-Ps in a similar manner to that to the SLT-V raters using the same voice samples. The CVT-Ps will undergo a training programme in the use of the CVT-STAR to improve inter-rater reliability [95, 96]. The mean rating of the four judges for each recording will be used as the data point for individual patients.

#### Aerodynamic measures: MPT

MPT is a simple and inexpensive aerodynamic voice parameter for measuring glottal competence and is expressed in seconds [93]. The patient is asked to inhale deeply and then sustain a steady /α/ vowel, as in farm, for as long as possible. The longest duration of the three consecutive attempts will be selected as the MPT measure for analysis. MPT will be measured from the time axis of the acoustic signal on the speech studio recording. The change from pre- to post-therapy value will be recorded and analysed with each subject acting as their own control to account for individual variation and the recognition of the significant difference between MPT values in men and women [100]. MPT can be used with caution as a measure of laryngeal dysfunction when inadequate glottal airway resistance is suspected and provides an indicator of the degree of ‘physiological support’ for speech [100]. However, it does not distinguish between inefficient glottal valving from reduced poor respiratory reserve and poor driving pressure of vocal fold vibration [100]. Values under 10 s are regarded as pathological. In a study of 8 patients with MTD using stretch-and-flow voice therapy by Watts et al. [59], there was a mean improvement in MPT from 12.36 ± 3.61 to 15.49 ± 4.33 (*p* = 0.14) with a significant clinical effect (medium effect size of *d* = 0.79). It was postulated that the improvement was due to improved control and coordination of the respiratory and laryngeal mechanisms associated with reduced physiological effort. It is hypothesised that MPT values will increase post-CVT therapy.

#### Secondary aim 1: is it feasible to perform a pilot study using CVT-VT administered by a CVT-P using telehealth?

The integrity of the protocol and rationale will be assessed under four main categories: process, resources, management and scientific [101]. The feasibility criteria, objectives, measures to be used and questions to be answered are outlined in (Table 3).

**Table 3:** Summary of feasibility criteria

OBJECTIVE	MEASURE	QUESTIONS
**PROCESS: FEASIBILITY OF THE PROCESSES THAT ARE KEY TO SUCCESS OF MAIN STUDY**		
Patients meeting eligibility criteria	• $\frac{\textrm{Number}\ \textrm{of}\ \textrm{patients}\ \textrm{meeting}\ \textrm{eligibility}\ \textrm{criteria}}{\textrm{Total}\ \textrm{number}\ \textrm{of}\ \textrm{patients}\kern0.5em \textrm{presenting}\ \textrm{with}\ \textrm{primary}\ \textrm{MTD}}\times 100\%$	• What is potential pool of patients with MTD?
**Concurrent acceptability:** Patients meeting eligibility criteria recruited	• $\frac{\textrm{Number}\ \textrm{of}\ \textrm{patients}\ \textrm{recruited}}{\textrm{Total}\ \textrm{number}\ \textrm{of}\ \textrm{patients}\ \textrm{meeting}\kern0.5em \textrm{eligibility}\ \textrm{criteria}}\times 100\%$ • Qualitative data on reasons for non-recruitment	• What is potential recruitment rate?
**Concurrent acceptability:** Recruitment rates against target	• $\frac{\textrm{Number}\ \textrm{of}\ \textrm{patients}\ \textrm{having}\ \textrm{at}\ \textrm{least}\ \textrm{one}\ \textrm{therapy}\ \textrm{session}}{\textrm{Total}\ \textrm{number}\ \textrm{of}\ \textrm{patients}\ \textrm{recruited}}\times 100\%$ • $\frac{\textrm{Number}\ \textrm{of}\ \textrm{patients}\ \textrm{having}\ \textrm{at}\ \textrm{least}\ \textrm{one}\ \textrm{therapy}\ \textrm{session}}{\textrm{Total}\ \textrm{number}\ \textrm{of}\ \textrm{patients}\ \textrm{recruited}\&\textrm{consented}}\times 100\%$ • Number of patients recruited in 6 month time period • Qualitative data on reasons for non-recruitment following consent • Qualitative data on reasons for non-progression to therapy following consent & recruitment	• What is potential recruitment to therapy rate? • Can recruitment be improved by modifying eligibility and exclusion criteria?
**Concurrent acceptability:** Total number of patients completing the study	• $\frac{\textrm{Number}\ \textrm{of}\ \textrm{patients}\ \textrm{completing}\ \textrm{the}\ \textrm{study}}{\textrm{Total}\ \textrm{number}\ \textrm{of}\ \textrm{patients}\ \textrm{recruited}}\times 100\%$ • Number of patients completing the study	• How many patients completed the study?
**RESOURCES: ASSESSING TIME AND RESOURCE PROBLEMS THAT CAN OCCUR DURING MAIN STUDY**		
Determining process time	• Qualitative data: administration of clinic appointments including patients contact • Qualitative data: Length of time to complete research clinic tasks	• How many patients can be seen per hour?
**Concurrent acceptability:** Adherence to protocol	• Dropout rates % • Qualitative data: on reasons for non-completion of treatment • $\frac{\textrm{Number}\ \textrm{of}\ \textrm{patients}\ \textrm{requesting}\ \textrm{further}\ \textrm{SLT}\ \textrm{therapy}\ \textrm{session}}{\textrm{Total}\ \textrm{number}\ \textrm{of}\ \textrm{patients}\ \textrm{recruited}\&\textrm{consented}}\times 100\%$ • Qualitative data: on reasons for requesting further therapy	• Does CVT-P address recruited MTD patients needs?
Patient costs	• Travel costs (median + range)	• What was the financial cost to the patient travelling to the research clinic appointments?
Missing data	• The amount of clinical outcomes data completed measured using the case report form at each time point (pre- and post-treatment) • Qualitative data: on reasons for missing data	• How much missing data is recorded and what where the reasons for this?
**MANAGEMENT: HUMAN & DATA MANAGEMENT PROBLEMS**		
Personnel/equipment/facility availability	• Qualitative data: Personnel/equipment/facility availability for research visits • Qualitative data: Equipment availability for patient • Qualitative data: issues with booking/attendance of therapy sessions	• Are there any issues with personnel/equipment/facility availability for research visits?
**Retrospective acceptability:** Teleheath	• Questionnaire using Likert scale (see Additional file 5: Patient/CVT-P goals and feedback questionnaire)	• Is the use of telehealth satisfactory for patients and the CVT-P?
**SCIENTIFIC: ASSESSMENT OF TREATMENT SAFETY, DOSE, RESPONSE, EFFECT, AND VARIANCE OF EFFECT**		
Study conduct	• Qualitative data: issues with documentation and data entry	• Is the Case Report Form adequate? • Is the Database adequate?
Safety	• Qualitative data of SLT-V observations of CVT-P sessions • Qualitative data: Feedback from any post-study SLT-VT • Reporting of adverse and serious adverse events • Qualitative data from sponsor monitoring visits	• Is CVT-VT safe to use healthcare intervention?
**Concurrent acceptability:** Number of CVT-VT sessions	• Average number of recorded CVT-VT sessions per patient • Qualitative data recording reasons for terminating sessions	• How many CVT-VT sessions were required on average? • Was this adequate?
**Retrospective acceptability:** Patient satisfaction with therapy	• Questionnaire using Likert scale: Descriptive statistics (see Additional file 5: Patient/CVT-P goals and feedback questionnaire)	• Was the patient satisfied with therapy received?
**Retrospective acceptability:** Achievement of patients goals	• Questionnaire using Likert scale: Descriptive statistics (see Additional file 5: Patient/CVT-P goals and feedback questionnaire)	• Did the patient achieve their pre-therapy goals following treatment?
**Retrospective acceptability:** CVT-P satisfaction with therapy	• Questionnaire using Likert scale: Descriptive statistics (see Additional file 5: Patient/CVT-P goals and feedback questionnaire)	• Was the CVT-P satisfied with therapy received?
**Retrospective acceptability:** Achievement of CVT-P goals	• Questionnaire using Likert scale: Descriptive statistics (see Additional file 5: Patient/CVT-P goals and feedback questionnaire)	• Did the CVT-P feel the pre-therapy goals agreed with the patient following treatment were met?
Primary outcome measure: Voice and vocal function	• Self-rated questionnaire (VHI): Inferential statistics: Difference in pre-post total scores , effect size	• Was the primary outcome measure for therapy achieved?
Secondary outcome measures: Throat symptoms	• Self-rated questionnaire (VTDS):Inferential statistics - Difference in pre-/post total scores , effect size	• Do these secondary outcome measures improve following therapy?
Secondary outcome measures: Aerodynamic measure	• MPT: Inferential statistics: Inferential statistics - Difference in pre-/post total scores , effect size	• Do these secondary outcome measures improve following therapy?
Secondary outcome measures: Objective voice measure & laryngeal vibratory pattern	• Acoustic/EGG measures: Inferential statistics - Difference in pre-/post total scores , effect size	• Do these secondary outcome measures improve following therapy?
Secondary outcome measures: Perceptual voice analysis	• Auditory-perceptual ratings (CAPE-V): Inter and intra-rater rating pre/post rating scores	• Do these secondary outcome measures improve following therapy?

#### Secondary aim 2: is CVT-VT acceptable to patients, CVT-Ps and SLT-VTs?

Acceptability is a multifaceted construct that reflects the extent to which people delivering or receiving a healthcare intervention consider it to be appropriate, based on anticipated or experiential cognitive and emotional response to the intervention [102]. Patients will be assessed for this feasibility outcome prospectively, concurrently and retrospectively and success will be rated using a traffic light system [43, 44].

#### Prospective acceptability

The project design was discussed with experienced SLT-Vs both at (*n* = 3) and external to the study site (*n* = 3). The CVT treatment protocols and assessment methods were reviewed and discussed with CVT teachers at the Complete Vocal Institute in Copenhagen (*n* = 10). Peer review was obtained by the Innovation Fund Denmark at the point of funding acceptance. The project was also discussed at an NUH Patient & Public Involvement (PPIE) ‘drop-in’ session in December 2021 and with 12 patients attending the Joint Voice clinic at NUH. All gave positive feedback and comments helped inform the final study design and protocol (see Additional file 4).

#### Concurrent acceptability

An important measure of acceptability of the study protocol to patients is recruitment and compliance with study protocol (see Table 3). Objective measures of behaviour, as indicators of acceptability, will be measured using:

a Number of eligible patients measured as a proportion of the total number of patients presenting during the study period with primary MTD recorded in the eligibility log
b Number of patients recruited to the trial as a proportion of those eligible, as recorded in the eligibility log
c Number of patients having at least one therapy session as a proportion of those recruited
d Number of patients having at least one therapy session as a proportion of those recruited and consented
e Number of patients completing the study as a proportion of those recruited, measured using the case report form at the end of the study
f The amount of clinical outcomes data completed measured using the case report form at each time point (pre- and post-treatment)
g Number of voice therapy sessions received by each patient measured using clinical notes at the end of the study and whether patients request additional SLT-VT at the end of the study

#### Retrospective acceptability

Retrospective assessment of will be limited to perceived effectiveness of treatment, including whether the goals of treatment were achieved, the satisfaction with the CVT-VT and use of telehealth. A non-validated patient questionnaire (see Additional file 5) will be used and the data obtained will be used to develop a more detailed questionnaire for use in future studies. A similar questionnaire will be administered to the treating CVT-P. The results will be reported using summary statistics. If further SLT-VT is requested by the patient qualitative data will be used to report what elements of the voice or vocal function had not been addressed. Acceptability of CVT-VT to the other important stakeholder, the SLT-Vs, will be assessed using a Likert scale (1) indicating their satisfaction with the response to therapy as administered by the CVT-P and (2) whether they would support the concept of a randomised controlled study of CVT-VT versus SLT-VT. The study results would be presented to 10 SLT-Vs at a meeting attended by specialist SLT-Vs.

#### Secondary Aim 3: does CVT-VT offer a new approach to improving the voice and vocal function compared to traditional SLT-VT methods?

A third feasibility outcome is to evaluate if, and how, CVT-VT differs from traditional SLT-VT methods of therapy. If CVT-VT offers a novel approach to management, it could be a useful additional tool for SLT-VTs in the management of MTD or provide a role for CVT-P’s in supporting SLT-Vs in the management of cohorts of MTD patients. Firstly, a qualitative assessment of the anonymised therapists’ sessional treatment records will be made using a preliminary organising framework and the initial codes will be identified and based on the ‘ingredients’ and ‘targets’ of the therapy methods applied during the treatment sessions. These codes will be used to assess the content of transcripts of the CVT-VT sessions using a qualitative thematic deductive content analysis approach. This will be based on the ‘ingredients’ and ‘targets’ outlined in the CVT methodology (see CVT intervention above) and by using the Rehabilitation Treatment Specification System (RTSS) [103] applied to SLT-V management of voice disorders [20, 29, 39]. This dual approach should allow easier direct comparison of ‘ingredients’ and ‘targets’ employed using both CVT and standard speech therapy terminology. This process will be aided by direct SLT-V observations of sampled CVT-VT sessions.

- Transcriptions of therapy sessions

Transcription of therapy sessions will be performed ad verbum*,* with an explicit focus on the detailing the ‘ingredients’ and ‘targets’ of the intervention techniques and exercises from both a CVT-VT and SLT-VT perspective [20, 29, 39]. A further potential value of the transcriptions is to provide supportive analyses for the documentation of the interventions and development of good clinical guidelines for working with CVT interventions i.e. how they should be explained, illustrated and exemplified and how they benefit the patient and their contextual usefulness.

Those patients who agree for their therapy sessions to be recorded will have these recordings made and stored in line with Trust-approved guidelines. The anonymised sessional recordings will then be transcribed and redacted to exclude any personal or identifiable information. The transcripts will be coded using the qualitative research management software NVivo based on the principles of template analysis [104], a commonly used thematic analytical framework allowing for a priori and crystalising themes in qualitative analyses. The main aim is to identify the differences and similarities of SLT-VT and CVT-VT approaches to therapy.

b) Observation of CVT therapy sessions by SLT-V

All patients will be asked to give specific consent for observation of their therapy sessions by the experienced study SLT-V. However, not all those who give consent will be observed for practical reasons, but the aim is to observe at least one patient through the 6 weeks of their therapy and sample a further six therapy sessions at different stages of their therapy. The observations will aid the SLT-V in identifying which ‘ingredients’ and ‘targets’ are being used and if they differ from traditional SLT-VT techniques. Further observations regarding the delivery methods, the type of feedback, progression rules and dosing of the ingredients will be made in line with the RTSS framework [20, 29]. An attempt will be made to outline the hypothesised ‘mechanisms of action’ and how both the ‘ingredients’ and ‘targets’ are linked to the patient ‘Aims’. This will enable a critical comparison of the type of techniques employed by the CVT-P to traditional SLT-VT techniques. In addition, observation of CVT-VT sessions provides a level of governance to ensure the patients goals are being met.

#### Sample size

The justification for the sample size is based on the objective of assessing feasibility [48]. In this study, the two main criteria are a change in the primary outcome measure, the VHI and recruitment. A previous representative study of SLT-VT in MTD of ten patients in the active treatment group resulted in a 50% reduction in VHI score. Recruiting ten patients in 6 months would therefore provide a good indication of potential improvement in the primary outcome measure and the ability to recruit an adequate number of patients in a 6 month time frame.

#### Progression criteria

Determining progression criteria is seen as an essential element in assessing the success of a feasibility study [44]. Using a traffic light system provides a method of defining targets for progression with green indicating ‘go’, amber ‘amend’ and red ‘stop’ [44]. Seven feasibility criteria have been identified that provide key indicators that will inform whether progression to a pilot randomised controlled study of CVT-VT versus SLT-VT. Firstly it is essential in this underpowered study that CVT-VT can improve the voice and vocal function i.e. VHI score. Although up to 50% change in VHI score is often seen in VHI scores for SLT-VT interventions [60], > 20% improvement seems adequate for a green outcome. Recruitment of > 9 patients who complete the study in 6 months would also indicate a satisfactory ‘green’ recruitment rate. From a patient and CVT-P perspective, a ‘green’ acceptability outcome of (‘very satisfied’ or ‘satisfied’) from the post-therapy questionnaire for at least 9 patients would be set as criteria (Table 4). For SLT-VTs, two criteria would be tested as ‘green’ criteria: (1) 8 or more out of 10 rating the outcome of the study as ‘very satisfied’ or ‘satisfied’ and (2) 8 or more out of 10 rating that they would be happy to support an RCT of CVT-VT versus SLT-VT.

**Table 4:** Summary of progression criteria using traffic light system

Aim	Green	Amber	Red
CVT-VT improves the VHI score	> 20% improvement in VHI score	10–20% improvement in VHI score	< 10% improvement in VHI score
A CVT-VT study is feasible to perform	> 9 patients completed pre- and post-therapy outcome assessments	8–9 patients completed pre- and post-therapy outcome assessments	< 8 patients completed pre- and post-therapy outcome assessments
CVT-VT is acceptable to patients	> 9 patients satisfied or very satisfied with therapy they have received	8–9 patients satisfied or very satisfied with therapy they have received	< 8 patients satisfied or very satisfied with therapy they have received
CVT-VT is acceptable to CVT-Ps	CVT-P satisfied or very satisfied with therapy delivered in > 9 patients	CVT-P satisfied or very satisfied with therapy delivered in 5–9 patients	CVT-P satisfied or very satisfied with therapy delivered in < 5 patients
CVT-VT is acceptable to SLT-VTs (1)	> 8 out of 10 SLT-VTs satisfied or very satisfied with the outcome of the study	> 5–8 out of 10 SLT-VTs satisfied or very satisfied with the outcome of the study	< 5 out of 10 SLT-VTs satisfied or very satisfied with the outcome of the study
CVT-VT is acceptable to SLT-VTs (2)	> 8 out of 10 SLT-VTs would support the concept of an RCT of CVT-VT vs SLT-VT based on the outcome of the study	> 5–8 out of 10 SLT-VTs would support progression to a RCT of CVT-VT vs SLT-VT based on the outcome of the study	< 5 out of 10 SLT-VTs would support progression to a RCT of CVT-VT vs SLT-VT based on the outcome of the study
Recruitment rate achieved	> 9 patients recruited in 6 months	8–9 patients recruited in 6 months	< 8 patients recruited in 6 months

### Data handling, analysis plan and statistical methods

We will record and report the participant flow according to CONSORT guideline and produce a CONSORT flowchart [105]. As a feasibility study, we expect to analyse recruitment and retention data using descriptive statistics involving both intention-to-treat and actual completed participant data. We shall report recruitment and retention figures together with reasons for loss of participants. An important part of this feasibility is also to assess whether six weekly treatment sessions provide adequate input to achieve the patient goals set at the outset of therapy and if not we shall report what was not addressed.

The supervising team and Sponsor (Nottingham University Hospitals Research and Development team) will monitor progress during treatment, consider any adverse effects and use that information to continue or halt the trial. Patients will be offered payment for travel costs for face-to-face research clinic appointments or to attend the outpatient clinic for video-linked therapy sessions if personal equipment is not adequate, but not for participation in the study.

Quantitative standard descriptive and inferential statistics methods will be applied to compare pre-and post-therapy measures (median + interquartile ranges with 95% CI). Statistical analyses of audio and EGG recorded data will be performed using Speech Studio (Laryngograph) and the SPSS Statistics package (Vers. 24.0.0.2 IBM Corporation, Chicago, IL).

VHI, VTDS and MPT will be reported using descriptive statistics (median + interquartile ranges with 95% CI) and the *P* values (0.5) and effect sizes calculated for the differences in pre- and post-treatment values using the Wilcoxon rank-sum test. Effect size together with the trial parameter data (i.e. recruitment, retention, follow-up and completion rate) will be used to determine the size of sample necessary to carry out a fully powered randomised controlled trial comparing CVT-VT to SLT-VT. For CAPE-V both descriptive statistics, the inter- and intra-rater reliability scores using Cohen’s Kappa will be reported for the individual parameters. For the acoustic and EGG measures descriptive summary statistics and pre- and post-therapy paired-samples *t* tests will be performed again using Wilcoxon rank-sum tests. The relationships between the results of the VHI, VTDS and MPT will be estimated by means of the Pearson *r* coefficient. Other non-validated questionnaires will be reported using descriptive statistics. Recruitment and retention data will be analysed using descriptive statistics.

### Ethics

Favourable ethical opinion for conduct has been granted by the East of England - Cambridge South Research Ethics Committee (Reference no. 22/EE/0047).

### Dissemination policies

The aim of dissemination will be to inform other speech and language therapists and CVT practitioners of the outcome of this approach of treating patients with MTD. This will be achieved through scientific conference presentations and workshops and feedback obtained to inform SLT-VT acceptability. A paper will be written for a peer-reviewed publication, and the results will be published using CONSORT extension guidelines for pilot and feasibility trials.

## Discussion

MTD is a common cause of voice problems but is a heterogeneous group of conditions causing varying degrees of functional limitation. The common feature for type I–III MTD is an imbalance of the three main mechanisms for voice production mainly breath support, laryngeal muscle tension and neck and pharyngeal muscle tension (hyper-constriction) affecting resonance. Traditional methods of treatment include a large range of direct voice therapy methods, often used in combination. Although widely employed successfully by SLT-Vs, many of the methods do not have a high level of evidence to support their use, and overlap in physiological aims. In addition, reducing constriction and producing a normal sounding voice do not always equate to improved voice function in demanding physical environments or social situations. CVT-VT is a well-defined structured approach that enables singers and other professional voice users/performers to produce their voice in a healthy manner for their vocal needs. CVT-VT has not been previously applied to a patient population, and this paper outlines the protocol for a feasibility study. The four aims of this study have been described i.e. (1) to demonstrate whether CVT-VT improves the voice and vocal function in patients with MTD as measured using the primary outcome measure (VHI); (2) whether a CVT-VT study is feasible to perform; (3) whether CVT-VT is acceptable to patients, CVT-Ps and SLT-VTs; and (4) whether CVT-VT offers a new approach to improving the voice and vocal function compared to traditional SLT-VT method. In addition, it will provide preliminary evidence on implementability of CVT-VT as a healthcare intervention by assessing the feasibility, acceptability and of this method as well as help develop criteria for future fidelity assessment [106]. Seven progression criteria for a randomised controlled pilot have been outlined using the traffic light system [44]. The protocol for this feasibility study has been developed according to the principles of good practice outlined by Lancaster et al. (2004) [50]. The SPIRIT 2013 checklist [107] for protocol development has been applied, and the study results will be reported against the checklist adopted from the CONSORT statement [46, 105].

## Supplementary Information

**Additional file 1.** Looking after your larynx advice leaflet.

**Additional file 2.** Outline of therapy sessions.

**Additional file 3.** CVT Speech Therapy Assessment Rating_CVT-STAR.

**Additional file 4.** Summary of patient survey.

**Additional file 5.** Patient/CVT-P goals and feedback questionnaire.

## Data Availability

Further details can be accessed at Muscle Tension Dysphonia Trial in Nottingham (Complete Vocal Technique Voice Therapy) | Clincosm. The final anonymised trial dataset as a result of this study will be available to other researchers on request from the corresponding author.

## Abbreviations

ANOVA: Analysis of variance
Ax: Amplitude of acoustic signal (dB)
CAPE-V: Consensus auditory-perceptual evaluation of voice
CI: Confidence interval
CVI: Complete vocal institute
CRF: Case-report form
CVT: Complete vocal technique
CVT-P: Complete Vocal Technique practitioner
CVT-VT: Complete Vocal Technique voice therapy
dB: Decibel
DPIA: Data Protection Impact Assessment
EGG: Electroglottography
ENT: Ear, Nose and Throat
EAI: Equal appearing interval
Fx: Fundamental frequency measured from EGG signal (Hz)
HNR: Harmonics to noise
HRA: Health Research Authority
HCRW: Health and Care Research Wales
Hz: Hertz
LTAS: Long-term average spectrum
MPT: Maximum phonation time
MTD: Muscle tension dysphonia
MTP: Muscle tension pattern
NNE: Normalised noise energy
NUH: Nottingham University Hospitals
PPIE: Patient and public involvement
RTSS: Rehabilitation Treatment Specification System
QMC: Queen’s Medical Centre
Qx: Contact quotient measured from EGG signal (%)
RFS: Reflux Finding Score
SD: Standard deviation
SLT: Speech and language therapy
SLT-V: Speech and language therapist specializing in voice
SLT-VT: Speech and language therapy voice therapy
VAS: Visual analog
VHA: Vocal hygiene advice
VHI: Voice Handicap Index
VT: Voice therapy
VTDS: Vocal Tract Discomfort Scale
