# Supplementary material for: A mixed-method feasibility study of the use of the Complete Vocal Technique (CVT), a pedagogic method to improve the voice and vocal function in singers and actors, in the treatment of patients with muscle tension dysphonia: a study protocol
Source: Pilot Feasibility Stud. 2023 May 24;9:88. doi: 10.1186/s40814-023-01317-y (PMC10206372; doi:10.1186/s40814-023-01317-y)
Supplement: Supplementary file 1 — Additional file 1. Looking after your larynx advice leaflet. [file 40814_2023_1317_MOESM1_ESM.docx]

| **Common irritants** | **Effects** | **How to help yourself** |
| --- | --- | --- |
| Smoking | Maybe any or all of the following: | Give up if you can. Cut down your exposure to smoky atmospheres. Seek professional help to quit |
| Dust/fluff | - Dryness | Where protective masks were issued at work and during house work if necessary |
| Pollen/animal hair | - Coughing or throat clearing | If affected by allergies to these or other things, use medicines only as directed |
| Fumes e.g. paint, glue, engine fumes | - Pain | Keep rooms ventilated |
| Household products e.g. polish, air freshener, perfume, hairspray | - Difficulty swallowing | Avoid breathing these products in use, and then leave the room while they settle |
| Medicines, especially decongestants, inhalers for asthma | - Swelling | Use only as directed by your doctor. Use the spacer and rinse your mouth out after inhaler use |
| Alcohol | - Changing natural secretions and   Throat | Keep to sensible limits. Consult your GP for information |
| Dehydration | - Tightness of the throat | Drink plenty of fluids, don’t wait to be thirsty. Aim for at least 1.5 to 2 L non—caffeinated drinks. Avoid excessive caffeine (in tea/coffee/cola) as this can drive a throat |
| Acid indigestion/heartburn |  | Consult your SLT/GP/pharmacist about medication to relieve symptoms. Avoid foods which aggravate the problem. Avoid eating within three hours of lying down. Raise the head of the bed so that the angle will assist downward flow of acid. See reflux advice sheet. |
| Dairy products |  | Avoid excessive intake if you find they give you a problem, such as catarrh or throat clearing |
| Dry atmosphere |  | Keep rooms ventilated. Use houseplants/bowls of water to humidify the atmosphere. Steam innovations help to relieve a dry throat reduce inflammation. |

**Looking after your larynx**
