# Supplementary material for: A mixed-method feasibility study of the use of the Complete Vocal Technique (CVT), a pedagogic method to improve the voice and vocal function in singers and actors, in the treatment of patients with muscle tension dysphonia: a study protocol
Source: Pilot Feasibility Stud. 2023 May 24;9:88. doi: 10.1186/s40814-023-01317-y (PMC10206372; doi:10.1186/s40814-023-01317-y)
Supplement: Supplementary file 2 — Additional file 2. Outline of therapy sessions. [file 40814_2023_1317_MOESM2_ESM.docx]

**Proposed outline of therapy session plans**

| Therapy session | Session plan |
| --- | --- |
| Therapy session 1 | 1. Go through patient goal setting questionnaire discussed with patient 2. Complete goal setting questionnaire part of CVT-P goal setting & CVT experience feedback questionnaire 3. Complete pre-treatment CVT Speech assessment form 4. Formulate management plan 5. Conduct therapy 6. Complete detailed Log of Therapist treatment form at end of session 7. Ensure all documents are saved on the Network shared drive 8. Give ‘homework’ to patient 9. Arrange next appointment with patient and book using DrDoctor |
| Therapy session 2-5 | 1. Review case details and documentation as necessary 2. Formulate management plan 3. Conduct therapy 4. Complete detailed Log of Therapist treatment form at end of session 5. Ensure all documents are saved on the Network shared drive 6. Give ‘homework’ to patient 7. Arrange next appointment with patient and book using DrDoctor |
| Therapy session 6 | 1. Review case details and documentation as necessary 2. Formulate management plan 3. Conduct therapy 4. Complete detailed Log of Therapist treatment form at end of session 5. Ensure all documents are saved on the Network shared drive 6. Inform JM of completion of Therapy session and so that Final research clinic appointment can be arranged 7. Complete original CVT-P goal setting & CVT experience feedback questionnaire 8. Complete end of therapy CVT Speech assessment form |
