# Supplementary figures and images for: A mixed-method feasibility study of the use of the Complete Vocal Technique (CVT), a pedagogic method to improve the voice and vocal function in singers and actors, in the treatment of patients with muscle tension dysphonia: a study protocol
Source: Pilot Feasibility Stud. 2023 May 24;9:88. doi: 10.1186/s40814-023-01317-y (PMC10206372; doi:10.1186/s40814-023-01317-y)

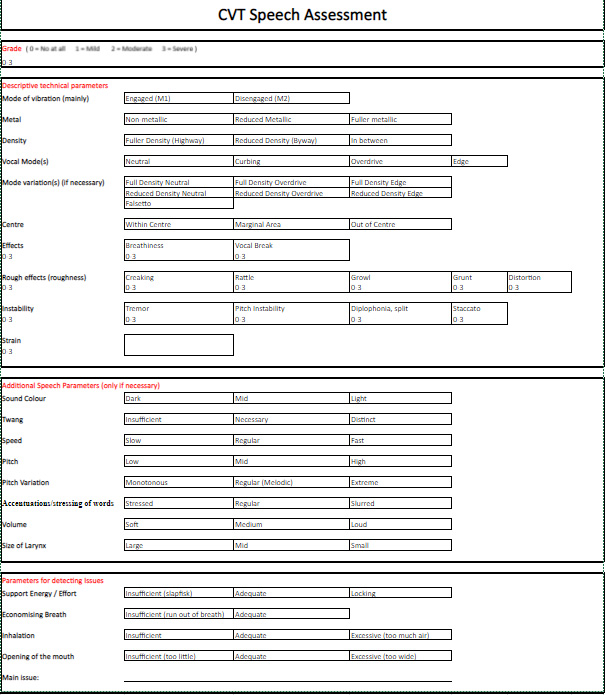


Additional Comments:

Supplement: Supplementary file 3 — Additional file 3. CVT Speech Therapy Assessment Rating_CVT-STAR. [file 40814_2023_1317_MOESM3_ESM.docx]
