# Supplementary material for: A mixed-method feasibility study of the use of the Complete Vocal Technique (CVT), a pedagogic method to improve the voice and vocal function in singers and actors, in the treatment of patients with muscle tension dysphonia: a study protocol
Source: Pilot Feasibility Stud. 2023 May 24;9:88. doi: 10.1186/s40814-023-01317-y (PMC10206372; doi:10.1186/s40814-023-01317-y)
Supplement: Supplementary file 4 — Additional file 4. Summary of patient survey. [file 40814_2023_1317_MOESM4_ESM.docx]

**Summary of Patient survey results to questionnaire regarding proposed study by patients attending Joint Voice clinic at NUH**

| **Question** | **Yes** | **No** | **Comments** |
| --- | --- | --- | --- |
| Having read through the information sheet, would you theoretically consider taking part in this study? | 10 | 2 | - No, because of the distance I have to travel - No, because I have spasmodic dysphonia |
| Do you think the aim of this pilot study, to test whether CVT-VT used in performers can help patients with MTD, is a good idea? | 12 | 0 |  |
| Do you have any concerns about patients having therapy with a specialist vocal coach (CVT-VT) rather than a Speech & Language therapist (SLT-VT) in this study? | 1 | 11 | - Yes, Are CVT-Ps as good as SLTs? |
| Do you have any concerns or comments about receiving your therapy using a video link? | 2 | 10 | - A small concern would be finding a quiet enough space to do it - I have found video links very stressful when my (spasmodic) dysphonia is bad |
| If you were a participant, would you be happy, have no strong opinion or be unhappy if the therapy sessions were recorded for more detailed analysis | 0 | 11 | (One patient did not answer) |
| If you were a participant, would you be happy, have no strong opinion or be unhappy if a specialist Speech and Language Voice Therapist observed the CVT Therapy sessions | 0 | 11 | (One patient did not answer) |
