# Supplementary material for: A mixed-method feasibility study of the use of the Complete Vocal Technique (CVT), a pedagogic method to improve the voice and vocal function in singers and actors, in the treatment of patients with muscle tension dysphonia: a study protocol
Source: Pilot Feasibility Stud. 2023 May 24;9:88. doi: 10.1186/s40814-023-01317-y (PMC10206372; doi:10.1186/s40814-023-01317-y)
Supplement: Supplementary file 5 — Additional file 5. Patient/CVT-P goals and feedback questionnaire. [file 40814_2023_1317_MOESM5_ESM.docx]

**Participant Number: …………...**

**Date: … … /… … …/… …**

**Research Clinic Visit (1 or 2) ……..**

**CVT Practitioner Experience Feedback Form:**

**Achievement of specific goals**

**(Kapsner-Smith, Hunter et al. 2015)**

1. **Therapy session 1:** Before starting treatment, please **indicate all of the problem(s)** you are trying to improve by placing a (X) in the grey box in the ’Goal set’ column on the left-hand side of the form in **Section A**
2. **After completion of therapy sessions:**
   1. Now you have completed treatment, how do you rate the achievement in improving the voice-related problems after the six weeks of therapy (Please indicate (X) in the box on the right-hand side of the form in **Section A**):
   2. Please also complete **Section B, C and D.**

**Section A: Goals of treatment**

| **Session 1** | | | **After completion of therapy sessions** | | | | | |
| --- | --- | --- | --- | --- | --- | --- | --- | --- |
| **Problem trying to improve** | **Goal set** | **Rank top 5** | **Problem resolved** | **Much improvement** | **Some improvement** | **No change or improvement** | **Problem worse** | **Problem a lot worse** |
| Able to sing again |  |  |  |  |  |  |  |  |
| Got voice back |  |  |  |  |  |  |  |  |
| Able to talk in a clear voice |  |  |  |  |  |  |  |  |
| Able to speak properly |  |  |  |  |  |  |  |  |
| Able to be heard on the phone |  |  |  |  |  |  |  |  |
| Able to be heard in day-to-day conversation |  |  |  |  |  |  |  |  |
| Able to shout |  |  |  |  |  |  |  |  |
| Able to make themselves heard in a noisy environment |  |  |  |  |  |  |  |  |
| No more voice breaks in speaking voice |  |  |  |  |  |  |  |  |
| No more voice breaks in singing voice |  |  |  |  |  |  |  |  |
| Not having to ask people to repeat themselves |  |  |  |  |  |  |  |  |
| Stop losing voice |  |  |  |  |  |  |  |  |
| Stop voice not cutting out |  |  |  |  |  |  |  |  |
| No longer feeling like something stuck or having discomfort in throat |  |  |  |  |  |  |  |  |
| To go through a working day without loss a voice |  |  |  |  |  |  |  |  |
| Not having to worry about voice |  |  |  |  |  |  |  |  |
| Feeling well, not sad because can't do it |  |  |  |  |  |  |  |  |
| Able to stop the pain when speaks |  |  |  |  |  |  |  |  |
| Other 1: |  |  |  |  |  |  |  |  |
| Other 2: |  |  |  |  |  |  |  |  |

**Please only complete this page at Visit 2**

**Participant Number: …………...**

**Date: … … /… … …/… …Research Clinic Visit (1 or 2) ……..**

**Section B: Experience of therapy**

| **Overall, how satisfied are you with:** | **Very satisfied** | **Satisfied** | **Neither satisfied or unsatisfied** | **Unsatisfied** | **Very unsatisfied** |
| --- | --- | --- | --- | --- | --- |
| the **therapy** you have delivered? |  |  |  |  |  |
| the **rapport** you developed with the patient? |  |  |  |  |  |
| the number of sessions of voice therapy? |  |  |  |  |  |
| the information you have given about the voice condition? |  |  |  |  |  |
| the information you have given about managing the voice problem? |  |  |  |  |  |

**Section C: Do you feel the patient needs a follow up appointment? Yes No**

**Section D: Using the video link**

Based on your experience of delivering the Voice therapy using a **video link**, please indicate (X) in the box below how much you agree or disagree with the following statements:

|  | **Strongly agree** | **Agree** | **Neither agree nor disagree** | **Disagree** | **Strongly disagree** |
| --- | --- | --- | --- | --- | --- |
| **Using a video link to deliver therapy was very satisfactory** |  |  |  |  |  |
| **The audio quality (*what you were able to hear*) during the majority of the sessions was very satisfactory** |  |  |  |  |  |
| **The video quality (*what you were able to see*) during the majority of the sessions was very satisfactory** |  |  |  |  |  |
| **I would be happy to deliver any voice therapy in the future via the video link** |  |  |  |  |  |
| **I would prefer to deliver any voice therapy in the future face-to-face** |  |  |  |  |  |

Please add any additional comments below:

Thank you.
